# Acute-onset multiple acyl-CoA dehydrogenase deficiency mimicking Guillain-Barré syndrome: two cases report

**DOI:** 10.1186/s12883-018-1221-2

**Published:** 2018-12-26

**Authors:** Daojun Hong, Yanyan Yu, Yuyao Wang, Yan Xu, Jun Zhang

**Affiliations:** 1 Department of Neurology, Peking University People’s Hospital, #11 Xizhimen South Avenue, Xicheng District, Beijing, 100044 China; 2 Department of Neurology, The first affiliated hospital of Nanchang University, Nanchang, China

**Keywords:** Multiple acyl-CoA dehydrogenase deficiency (MADD), Guillain-Barré syndrome (GBS), ETFDH, Phenotype, Differential diagnosis, Acute onset

## Abstract

**Background:**

Multiple acyl-CoA dehydrogenase deficiency (MADD) showed great clinical heterogeneity and poses a challenge to diagnosis. Guillain-Barré syndrome (GBS) is an acute-onset autoimmune-mediated peripheral neuropathy. However, no patients of acute-onset MADD mimicking the GBS phenotype are reported previously.

**Case presentation:**

Two patients displayed acute-onset limb weakness, areflexia, and length-dependent sensory disturbances, which clinically indicate the diagnosis of GBS, but electrophysiological and cerebrospinal fluid results threw doubtful points to the initial diagnosis. The muscle biopsy showed lipid storage disorder; and compound heterozygous mutations in the electron transfer flavoprotein dehydrogenase (ETFDH) gene were found in the two patients through targeted next generation sequencing, which provided the definite diagnostic evidences of late-onset MADD. Muscle weakness was quickly improved by riboflavin supplementation, but sensory disturbances required a long-term treatment.

**Discussion:**

The present two cases have demonstrated that MADD can mimic GBS. Taking into consideration the significant differences of therapeutic regimen and prognosis, MADD should be included in the differential diagnosis of GBS.

## Background

Guillain-Barré syndrome (GBS), also known as Landry’s paralysis, is an acute or subacute polyradiculoneuropathy [1]. It has been believed that GBS may not be a single entity, but a group of immune-mediated neuropathies with pathogenesis associated with endoneurial inflammation of spinal nerve roots, distal nerve segments, and potential nerve entrapment sites [2]. The commonly recognized variants of GBS are acute inflammatory demyelinating polyneuropathy (AIDP), acute motor axonal neuropathy, acute motor sensory axonal neuropathy, and Miller-Fisher syndrome. AIDP is the most prevalent form and accounts for 70–90% of GBS cases [3]. The main clinical features of GBS include acute-onset generalized limb weakness, limb paraesthesias, and relative or complete areflexia. The typically symptomatic pattern exhibits an ascending flaccid paralysis evolving over a few days to a few weeks. In some situations, a history of antecedent respiratory or gastrointestinal infection or vaccination can be identified [4]. Respiratory failure and oropharyngeal weakness may require ventilator assistance in about one-third of hospitalized patients making it as a disease of vital importance for early management [5]. However, it is difficult to make a rapid and correct diagnosis of GBS sometimes, because some rare differential cases can mimic the clinical presentations of GBS [6–8].

Multiple acyl-CoA dehydrogenase deficiency (MADD; OMIM, 231680) is an autosomal recessive inherited metabolic disorder mainly caused by the defects of electron transfer flavoprotein ubiquinone oxidoreductase (ETF:QO) complex encoded by the ETF dehydrogenase (*ETFDH*) gene, alpha ETF (*ETFA*) gene, and beta ETF (*ETFB*) gene [9]. The phenotype of late-onset MADD is highly variable and mainly characterized by fluctuating muscle weakness, vomiting, hypoglycemia, metabolic acidosis, and hepatomegaly usually preceded by metabolic stress [10]. In the recent decade, a lot of patients with late-onset MADD have been reported in Chinese population who mainly presented with proximal muscle weakness, exercise intolerance, myalgia, and dramatic riboflavin responsiveness. Therefore, this clinical phenotype is also named riboflavin responsive MADD (RR-MADD) [11].

Recently, Wang et al. reported that six patients with late-onset MADD caused by *ETFDH* mutations presented with chronic sensory disturbances besides relatively acute muscle weakness, suggesting that sensory neuropathy might be involved in the clinical spectrum of late-onset MADD [12]. However, no cases of late-onset MADD with acute-onset motor-sensory symptoms mimicking GBS were reported previously. In this report we described two Han Chinese patients initially presenting with acute-onset limb weakness, areflexia, and length-dependent sensory disturbances, which clinically pointed to the diagnosis of GBS, but a diagnosis of late-onset MADD was finally made.

## Case presentation

### Case 1

The patient was a 22-year-old man who came from a non-consanguineous family. Before he was referred to our department, he had a history of antecedent influenza 10 days ago. He started to have progressive limb weakness and numbness 5 days ago. He also complained of persistent soreness in both inferior calves and numbness in the distal limbs. The symptoms gradually worsen, and caused virtually bed bound. Neurological examinations revealed a length-dependent decrease of touching, temperature, pain, and vibration sensations below the knee and the wrist joints. Muscle strength was grade 2−/5 (Medical Research Council scales) in the proximal lower limbs, grade 4/5 in the distal lower limbs, grade 4−/5 in the upper limbs, and grade 3/5 in the neck flexion. The cranial nerves were intact. Deep tendon reflexes were not elicited.

Serum creatine kinase (CK) was 5809 IU/L (normal 1–171 IU/L). Blood count, blood biochemistry, inflammatory tests, thyroid hormones, serum vitamin B12 and folic acid were in normal limits. The panel of anti-ganglioside antibodies including GQ1b, GT1b, GD1b, GD1a, GM2, and GM1 was negative. Cerebrospinal fluid (CSF) results were normal at 6 days after the onset of disease. Blood acylcarnitine profile before treatment revealed a combined elevation of short-, medium-, and long-chain acylcarnitines. Urine organic acid analysis indicated an increase of multiple metabolic acids.

Motor nerve conduction velocity (MNCV) showed that a borderline decrease of compound muscle action potentials (CMAP) was recorded in the bilateral median, bilateral ulnar, and left peroneal nerves (Table 1). Sensory NCV showed normal potentials can be recorded in both sural nerves and median nerves. The latency of H reflexes and F waves were normal in all nerves tested. Needle electromyogram showed mildly decreased motor unit action potentials (MUAP) in the right gastrocnemius muscle and anterior tibialis muscle.

**Table 1:** Electrophysiological studies of MADD in the two patients

Motor nerve		Case 1			Case 2		
		MNCV(m/s)	dL (ms)	CMAP (mV)	MNCV(m/s)	dL (ms)	CMAP (mV)
L Median	E-W	74(≥50.0)	–	7.2(≥5.0)	54	–	13.0
	W-APB	–	3.3(≤4.2)	8.5(≥5.0)	–	3.3	13.8
R Median	E-W	67	–	5.3	56	–	16.1
	W-APB	–	3.4	5.8	–	3.0	16.9
L Ulnar	E-W	66(≥53.0)	–	7.6(≥5.5)	53	–	11.0
	W-ADM	–	2.1(≤4.2)	9.5(≥5.5)	–	2.5	12.4
R Ulnar	E-W	68	–	8.1	61	–	10.7
	W-ADM	–	2.0	8.5	–	2.2	11.0
L Peroneal	FH-A	47(≥38.0)	–	3.7(≥3.0)	43	–	2.6
	A-EBD	–	3.0(≤6.1)	5.1(≥3.0)	–	3.8	3.3
R Peroneal	FH-A	58	–	10.3	50	–	3.3
	A-EBD	–	3.7	12.5	–	4.6	3.7
L Tibial	PF-A	51(≥35.0)	–	12.0(≥3.0)	50	–	13.6
	A-AA	–	4.8(≤6.1)	13.9(≥3.0)	–	4.8	165
R Tibial	PF-A	56	–	11.4	52	–	11.5
	A-AA	–	4.4	11.2	–	5.3	12.8
Sensory nerve		SNCV (m/s)		SNAP (uV)	SNCV (m/s)		SNAP (uV)
L-Median	IIF-W	59(≥50.8)		14.4(≥8.0)	35		1.5
R-Median	IIF-W	57		11.9	48		1.1
L-Sural	A-SURA	42(≥41.9)		15.5(≥7.0)	NR		NR
R-Sural	A-SURA	45		26.3	NR		NR
F wave		F-Lat(ms)			F-Lat(ms)		
L-Median		29.5(≤32.0)			27.31		
R- Median		27.62			27.0		
H Reflex		H-Lat(ms)			H-Lat(ms)		
L Tibial		28.0(< 40.0)			32.89		
R Tibial		28.0			31.26		

*MNCV* motor nerve conduction velocity, *CMAP* compound motor action potential, *dL* distal motor latency, *SNCV* sensory nerve conduction velocity, *SNAP* sensory nerve action potential, *L* left, *R* right, *E* elbow, *W* wrist, *APB* abductor pollicis brevis, *ADM* abductor digiti minimi, *FH* fibula head, *A* ankle, *EDB* extensor digitorum brevis, *PF* popliteal fossa, *AA* abductor allucis, *IIF* second finger, *SURA* sural, *Lat* latency, *NR* no recorded. Normal values are given in brackets

### Case 2

The patient was a 55-year-old man who came from a non-consanguineous family. He suddenly began to have muscle weakness in both lower limbs; meanwhile he felt numbness of the distal lower limbs. The weakness quickly ascended to upper limbs 2 days later and then progressed into difficulty of swallowing 3 days later. He also complained of tightness around the waist and abdomen, but the bladder function was normal. Muscle strength was grade 4/5 in the foot dorsiflexors, grade 5/5 in the plantar flexion, grade 2/5 in the proximal lower limbs, grade 4/5 in the hand gripping, and grade 3/5 in the proximal upper limbs. The sensations of pain, vibration, and joint position perception reduced below the knee. Deep tendon reflexes were not elicited in the lower and upper limbs.

Serum CK was 334 IU/L. Blood count, blood biochemistry, inflammatory indexes, thyroid hormones, serum vitamin B12 and folic acid were in normal limits. The panel of anti-ganglioside antibodies including GQ1b, GT1b, GD1b, GD1a, GM2, and GM1 was negative. Laboratory panels of CSF were normal at 5 days after the onset of disease. Spinal MRI was normal. Blood acylcarnitine profile before treatment revealed a multiple increase of short-, medium-, and long-chain acylcarnitines. Urine organic acid analysis showed a significant elevation of 2-hydroxyglutaric acid and 2-hydroxyadipic acid.

MNCV of the case 2 revealed decreased amplitudes of CMAP in both peroneal nerves, but other nerves were intact (Table 1). Sensory NCV showed significant impairments in nerves tested. The latency of H reflexes and F waves were normal in all nerves tested. Needle electromyogram of gastrocnemius muscle showed a little short duration and low amplitude MUAP.

**Fig. 1:**
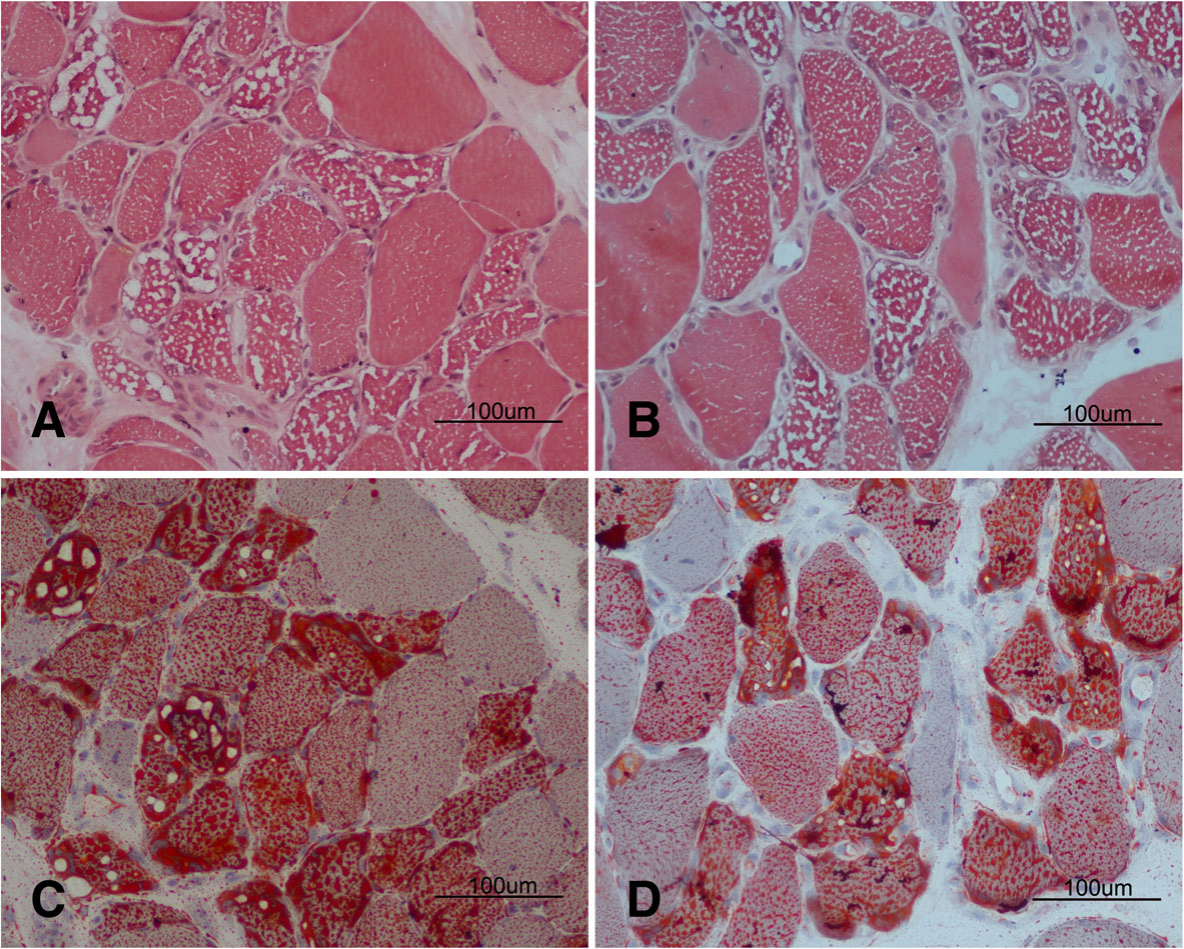
Pathological changes in the muscle specimen. Hematoxylin-eosin stain revealed numerous small vacuoles appearing in most muscle fibers of case 1 (**a**) and case 2 (**b**). Oil red O stain showed massive lipid droplets depositing in the corresponding vacuoles of case 1 (**c**) and case 2 (**d**)

Muscle biopsies were conducted at the right biceps brachii in the two patients. The muscle specimens exhibited similar pathological changes. Most myofibers were filled with numerous small vacuoles, but without significant variations of fiber diameter or proliferation of connective tissue (Fig. 1a and b). The lipid droplets were accumulated in the fibers with vacuoles (Fig. 1c and d), especially affecting the type I fibers. Nicotinamide adenine dinucleotidetetrazolium reductase (NADH-TR) stain revealed many dark particles in the fibers with numerous lipid droplets. A few fibers with negative cytochrome *c* oxidase (COX) were observed in the two cases. Neurogenic patterns such as grouping of angular atrophic fibers or target-like fibers were not observed in the acid or alkaline ATPases stain.

**Fig. 2:**
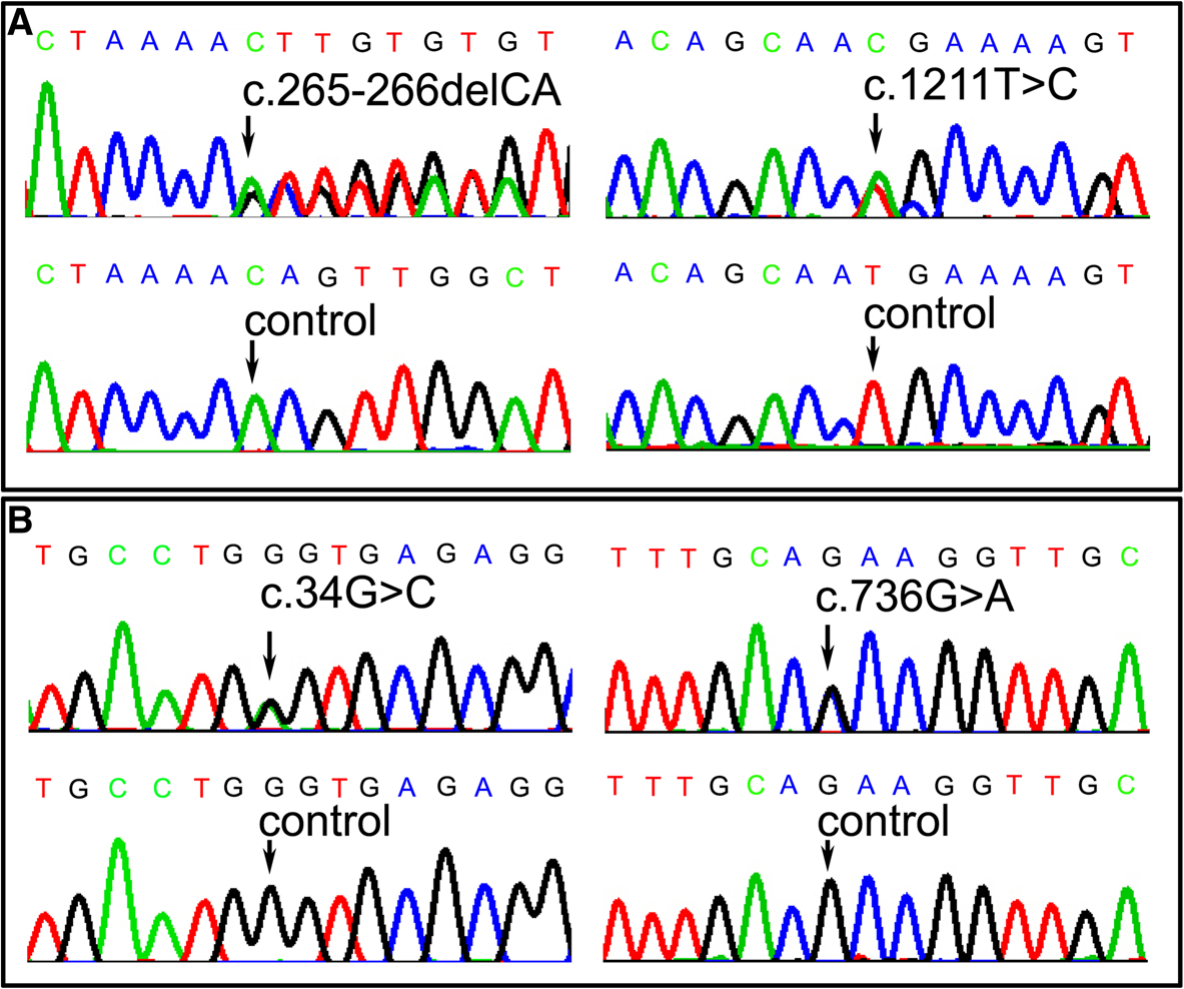
The chromatogram of *ETFDH* variants. The compound heterozygous mutations c.34G > C and c.1211 T > C in case 1 (**a**), and compound heterozygous mutations c.736G > A and c.1454C > G in case 2 (**b**)

Genetic test was performed in the patients through targeted next generation sequencing (NGS) after informed consents were written. The NGS was conducted on selected subjects using Agilent SureDesign Panel kits for inherited myopathy and inherited peripheral neuropathy. Genetic sequencing disclosed compound heterozygous mutations: c.265-266delCA and c.1211 T > C (p.M404 T) in the case 1 (Fig. 2a); c.34G > C (p.A12P) and c.736G > A (p.E246K) in the case 2 (Fig. 2b). The variants co-segregated with their parents: c.265-266delCA was from the mother and c.1211 T > C was from the father; c.34G > C was from the father and c.736G > A was from the mother. The variants c.736G > A and c.265-266delCA were not found in the 1000 genomes database, ExAC database, and gnomAD database, but the variants c.34G > C and c.1211 T > C had a very low allele frequency (Table 2). A homology search in different species demonstrated that the amino acid at residues 12, 246, and 404 were highly evolutionarily conserved, respectively. The variants were predicted to be damaging by several in silico tools (Table 2). The pathogenicity of variants was evaluated according to the American college medical genetics and genomics (ACMG) criteria (Table 2). No other causative mutations associated with metabolic myopathies or inherited neuropathies were found in the target gene kits including the *ETFA*, *ETFB*, flavin adenine dinucleotide synthetase 1 (*FLAD1*), and solute carrier family 25 member 32 (*SLC25A32*) genes.

**Table 2:** The pathogenic analysis of variants in the *ETFDH* gene

Novel variants	Protein change	gnomAD (frequency)	PolyPhen-2	SIFT	Mutation Taster	ACMG criteria
c.34G > C	p.A12P	0.000003978	1.00	0.00	disease causing	likely pathogenic
c.265_266delCA	p.Q89Vfs*5	0	–	–	disease causing	pathogenic
c.736G > A	p.E246K	0	0.959	0.01	disease causing	pathogenic
c.1211 T > C	p.M404 T	0.00000796	0.989	0.00	disease causing	pathogenic

*gnomAD* Genome aggregation database, *SIFT* sorting tolerant from intolerant, *ACMG* American college medical genetics and genomics criteria

*Indicates at which codon position the new reading frame ends in a stop codon

The two patients were initially treated with riboflavin (150 mg/d), L-camitine (2 g/d), and coenzyme Q10 (150 mg/d). One week later, limb weakness improved dramatically, and muscle strength nearly recovered 4 weeks later. The level of CK also returned to normal limits. The sensory disturbances showed no improvement; even the tightness around waist and abdomen in case 2 became worse than ever 4 weeks later. However, the patients reported significant improvement of paraesthesias after long-term administration of riboflavin (30 mg/d), CoQ10 (100 mg/d), and cobalamine (500μg/d) for 12 months.

## Discussion and conclusion

The two patients clinically showed acute-onset muscle weakness and limb numbness, and the patient’s condition quickly deteriorated to be bulbar paralysis or bedridden. Therefore, the patients were initially suspected as Guillain-Barre syndrome. However, when cerebrospinal fluid and electrophysiological results threw doubtful points to the initial diagnosis [4, 6], hyperCKmia gave the diagnostic clue to myopathy. Finally, muscle biopsy and genetic screening provided the definite diagnostic evidences of late-onset MADD caused by mutations in the *ETFDH* gene.

Late-onset MADD usually manifests as fluctuating weakness, but sometimes chronic or sub-acute length-dependent sensory neuropathy can be observed in MADD patients [12]. However, the patients in this report presented with acute-onset muscle weakness and limb paraesthesias resembling symptoms of GBS, because the main clinical features of GBS included monophasic disease course, symmetrical limb weakness, hypo/areflexia, and distal limb paraesthesias. Given the significant differences of therapeutic regimen and prognosis, late-onset type of MADD with acute onset should be considered as a differential diagnosis of GBS in the clinical workflow [5, 13].

MADD as a metabolic myopathy can be triggered at several days after experiencing stress conditions such as malnutrition, labored work, childbirth, and infections [14]. However, GBS usually presents with symptoms at a couple of weeks after a history of respiratory or gastrointestinal infections or vaccination [2]. Therefore, the differences of antecedent course and triggers can give valuable clues to differentiate the two diseases.

Different electrodiagnostic criteria have been proposed to diagnose GBS in the recent decades [6]. Cerebrospinal fluid analysis and electrodiagnostic testing may be normal in the early phase of GBS, but F-wave and H-reflex are the early sensitive means to detect the abnormalities of spinal nerve roots [15]. The amplitude of motor NCV showed a borderline decrease in some nerves of case 1, it might indicate a possibility that a GBS had superimposed to underlying MADD, but it might also be the consequence of inborn metabolic dysfunctions. The neurophysiological results indicated a length-dependent axonal sensory neuropathy without abnormalities of nerve roots. Cerebrospinal fluid analysis and anti-ganglioside antibodies were normal in the cases. In addition, the treatment response also rebutted the possibility of GBS superposition. Taken together, abovementioned findings let us doubt the diagnosis of GBS, so the different neurophysiological patterns are useful to differentiate between the two diseases.

The level of serum CK can vary from normal to hundreds of times in patients with late-onset MADD, but it is only elevated to several times in a little part of GBS patients [16]. The significant hyperCKmia in the case 1 gives us an important clue to think about myopathy. Because we have the lesson from the first case, we successfully avoid a misdiagnosis of the second case, even if the level of CK is only elevated to two times. Therefore, if a patient presents with proximal muscle weakness and distal sensory disturbance, as well as hyperCKmia, MADD should be considered as one of candidate diagnosis.

Our observation indicated that riboflavin formula could quickly resolve the muscle weakness, as reported by others [17], but the symptoms of sensory disturbance had no improvement in the short-term riboflavin treatment. The therapeutic response indicated that the damage of sensory nerves might be intractable to riboflavin supplementation in the two cases. Zebrafish model with defect of ETF:QO displayed a low response to touch stimulation and a disorganized sensory axonal tract with hypomyelination [18]. Marked neurite shortening were also observed in the cells expressing the *ETFDH*-mutant, but the neurite shortening could be restored by mitochondrial cofactor supplementation with carnitine, riboflavin, or coenzyme Q10 [19]. Intriguingly, sensory disturbances in the two cases showed significant improvements after long-term administration of riboflavin, coenzyme Q10 and vitamin B12, which attested the efficiency of mitochondrial cofactor supplementation to the neurite growth impaired by *ETFDH*-mutants.

ETF-QO structurally consists of a flavin adenine dinucleotide binding region, a ubiquinones binding region, and a 4Fe4S cluster [20]. ETF:QO is an important electron transfer protein on the intra-mitochondrial membrane. Riboflavin might help the folding, assembly, and stability of defects mutant ETF-QO, and recover the catalytic activity of flavoproteins. Those can well explain the reason of lipid storage myopathy, but the detailed pathomechanism how mutant ETF-QO impairs sensory nerves is still unclear. The variants in the two patients locate in hotspot FAD binding domain, so no specific relationship of genotype-phenotype can be identified [21]. The acute-onset course may be associated with individual and environmental factors.

In conclusion, the present two cases demonstrated that *ETFDH* gene variants can cause acute-onset proximal muscle weakness and distal sensory disturbance. Taking into consideration the significant differences of therapeutic regimen and prognosis, MADD should be included in the list of differential diagnosis for GBS.

## Abbreviations

AIDP: Acute inflammatory demyelinating polyneuropathy
CK: Creatine kinase
CMAP: Compound muscle action potentials
COX: Cytochrome *c* oxidase
ETF:QO: ETF ubiquinone oxidoreductase
ETFDH: Electron transfer flavoprotein dehydrogenase
GBS: Guillain-Barré syndrome
MADD: Multiple acyl-CoA dehydrogenase deficiency
MNCV: Motor nerve conduction velocity
MUAP: Motor unit action potentials
NADH-TR: Nicotinamide adenine dinucleotidetetrazolium reductase
